# High operating temperature in V-based superconducting quantum interference proximity transistors

**DOI:** 10.1038/s41598-017-09036-0

**Published:** 2017-08-18

**Authors:** Nadia Ligato, Giampiero Marchegiani, Pauli Virtanen, Elia Strambini, Francesco Giazotto

**Affiliations:** 1 NEST Istituto Nanoscienze-CNR and Scuola Normale Superiore, Piazza S. Silvestro 12, I-56127 Pisa, Italy; 2 Dipartimento di Fisica dell’Università di Pisa, Largo Pontecorvo 3, I-56127 Pisa, Italy

## Abstract

Here we report the fabrication and characterization of fully superconducting quantum interference proximity transistors (SQUIPTs) based on the implementation of vanadium (V) in the superconducting loop. At low temperature, the devices show high flux-to-voltage (up to 0.52 mV/Φ_0_) and flux-to-current (above 12 nA/Φ_0_) transfer functions, with the best estimated flux sensitivity ~ 2.6 *μ*Φ_0_/(Hz)^1/2^ reached under fixed voltage bias, where Φ_0_ is the flux quantum. The interferometers operate up to *T*_bath_ $\simeq$ 2 K, with an improvement of 70% of the maximal operating temperature with respect to early SQUIPTs design. The main features of the V-based SQUIPT are described within a simplified theoretical model. Our results open the way to the realization of SQUIPTs that take advantage of the use of higher-gap superconductors for ultra-sensitive nanoscale applications that operate at temperatures well above 1 K.

## Introduction

Currently, the possibility to control electrical^1, 2^ and thermal transport^3, 4^ in hybrid superconducting systems has generated strong interest for nanoscale applications, including metrology^5, 6^, quantum information^7^, quantum optics^8^, scanning microscopy^9^, thermal logic^10, 11^ and radiation detection^12^.

In this scenario, the superconducting quantum interference proximity transistor (SQUIPT)^13, 14^ represents a concept of interferometer which shows suppressed power dissipation and extremely low flux noise comparable to conventional superconducting quantum interference device (SQUID)^2, 15^. A SQUIPT consists of a short metal wire (i.e., a weak-link) placed in good electric contact with two superconducting leads defining a loop and a metal probe tunnel-coupled to the nanowire. As a consequence of the wire/superconductor contacts, superconducting correlations are induced locally into the weak-link through the proximity effect^16–18^. This results in a strong modification of the density of states (DOS) in the wire, where a minigap is opened^19^. The key factor of the device is the possibility to control the wire DOS and thus the electron transport through the tunnel junction, by changing the superconducting phase difference *φ* across the wire-superconductor boundaries through an applied magnetic field which gives rise to a flux Φ piercing the loop area.

The transparency of the nanowire/superconductor contacts plays a key role in the device sensitivity, because the induced minigap in the wire DOS is highly sensitive to the interface transmissivity, and decreases as the contacts become more opaque^20^. In this sense, it is convenient to realize SQUIPTs where the nanowire and the loop are made of the same superconducting material due to the higher quality of the contacts interface as well as the simpler fabrication process. Recently, the features of fully superconducting Al-based SQUIPTs have been theoretically and experimentally investigated^21, 22^.

So far, SQUIPT configurations show a wide use of Al as the superconducting material^13, 23, 24^. Its popularity is mainly due to the simple and extensive know-how of Al film deposition, and due to its high-quality native oxide which allows the realization of excellent tunnel barriers through room-temperature oxidation. However, the low value of the Al critical temperature (*T*_c_ = 1.2 K) is synonymous with low operation temperatures, and the use of superconducting metals with higher *T*_c_ is greatly desired for technological applications. The use of elemental metals such as vanadium (V) and niobium (Nb) is technologically demanding but would enable the possibility to significantly extend the SQUIPT working temperature. Nb has a high *T*_c_ = 9.2 K, but also a high melting point that requires more complex nanofabrication processes^25, 26^. Vanadium is a group-*V* transition metal, such as Nb, with a bulk *T*_c_ = 5.4 K, but its lower melting point allows easier evaporation^27–30^.

An essential requirement for an optimal phase bias of the SQUIPT device is that the kinetic inductance of the superconducting ring, ${L}_{{\rm{kin}}}^{{\rm{R}}}$, be negligible compared to that of the nanowire, ${L}_{{\rm{kin}}}^{{\rm{NW}}}$, i.e. ${L}_{{\rm{kin}}}^{{\rm{NW}}}/{L}_{{\rm{kin}}}^{{\rm{R}}}\gg 1$ ^31^. This condition makes using refractory metals as the ring material less favorable, due to the typically higher values of their resistivity (see Supplementary Information)^32–34^.

Here we report the fabrication and characterization of V-based SQUIPTs realized with a V-Al bilayer ring. On the one hand the V implementation on top of an Al-SQUIPT ring allows us to extend the maximal operating temperature up to $T\sim 2$ K, granting a significant improvement of the operating temperature range with respect to early Al-based SQUIPTs. On the other hand the Al layer acts as a “shunt inductor” to ensure a low value of the ${L}_{{\rm{kin}}}^{{\rm{R}}}$ for an optimal phase bias of the device. At low temperature our interferometers show good magnetic sensing performance, with a maximum flux-to-voltage transfer function of $\sim 0.5\,{\rm{mV}}/{{\rm{\Phi }}}_{0}$ and a maximum flux-to-current transfer function of $\sim 12\,{\rm{nA}}/{{\rm{\Phi }}}_{0}$, where ${{\rm{\Phi }}}_{0}\simeq 2.068\times {10}^{-15}\,{\rm{Wb}}$ is the flux quantum. The maximum flux sensitivity $\sim 2.6\,\mu {{\rm{\Phi }}}_{0}/\sqrt{{\rm{Hz}}}$ is obtained under optimal voltage bias.

The Section Results is organized as follows. In the Subsection Interferometers design we briefly discuss the design and the fabrication of the device. The electric characterization at low temperature is presented in the Subsection Transport spectroscopy. The Subsection Magnetic sensing performance is devoted to the magnetometric behaviour at low temperature, with an evaluation of the transfer functions and the flux sensitivity. In the Subsection Impact of the bath temperature the temperature evolution of the interferometers features is discussed.

## Results

### Interferometers design

**Figure 1:**
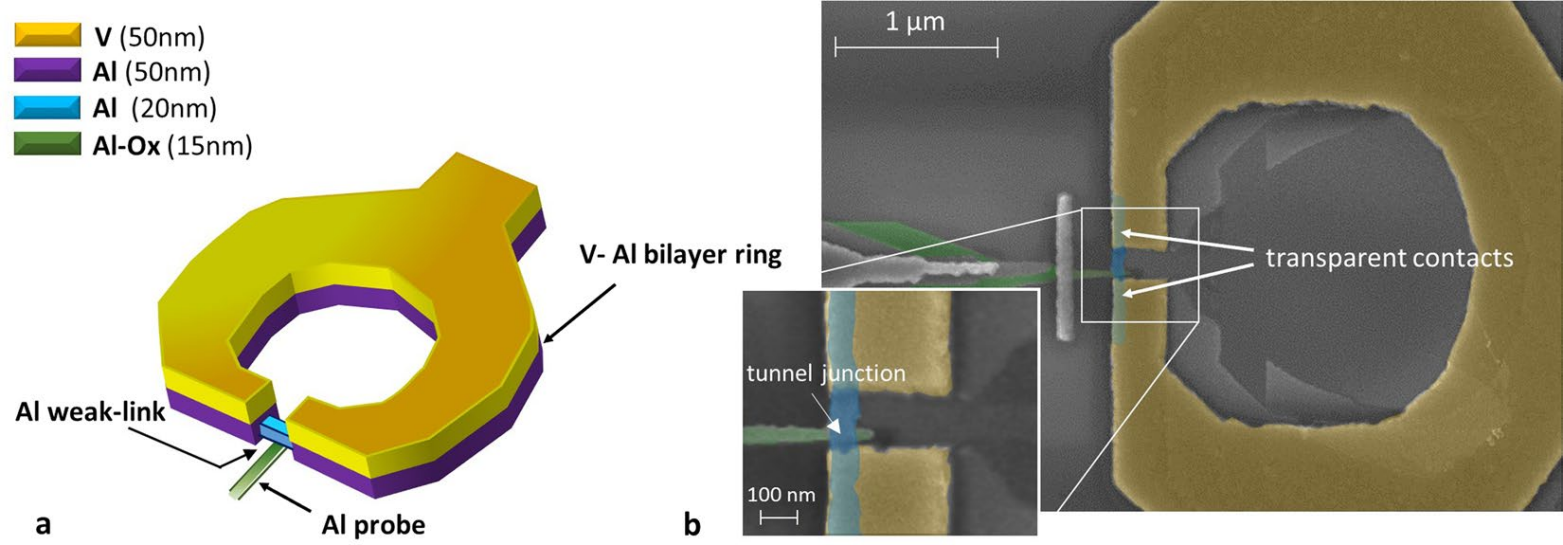
Design and scanning electron micrograph (SEM) of the V-based SQUIPT. (**a**) Sketch of the V-based SQUIPT. An Al nanowire is embedded into a V-Al ring and an Al probe is tunnel-coupled to the middle of the wire. (**b**) False-color SEM of sample-A with an enlarged view centered on the junction region. In the SEM image the passive metal replicas deriving from the three-angle shadow-mask evaporation are also visible.

The SQUIPT concept design [see Fig. 1(a)] is based on an Al nanowire embedded in a thick V-Al bilayer ring. Furthermore, an Al probe electrode is tunnel-coupled to the Al wire. The V layer deposited on top of the Al ring allows to increase the size of the minigap induced in the nanowire DOS, without compromising the Al/AlO_x_/Al junction quality. The Al layer was first deposited to insure the quality of the interface between the wire and the ring. The loop geometry of the superconducting electrode makes it possible to change the phase difference *φ* across the superconducting wire by applying an external magnetic field, due to the flux quantization. The choice of a thick layer for the superconducting ring is a necessary condition: (i) to reduce the inverse proximity effect of the Al wire on the bilayer ring and (ii) to decrease its normal-state resistance, and thus its kinetic inductance, thereby allowing a good phase biasing of the weak-link.

Interferometers are realized by electron-beam lithography (EBL) combined with three-angle shadow-mask evaporation (see Methods). Figure 1(b) shows a false-color scanning electron micrograph (SEM) of a typical V-based SQUIPT with a magnification of the weak-link zone. A crucial step in the processing is the vanadium deposition. Electron-beam evaporation of a refractory superconductor material such as V requires some special considerations. If no precautions are taken, the heating of the substrate damages the resist layer with the consequent metal-pattern deterioration. In this regard, Table 1 lists the characteristic parameters for all samples and demonstrates the excellent reproducibility achieved as a consequence of the fabrication process optimization.

**Table 1:** Parameters of different SQUIPT samples. *L* is the interelectrode spacing, *w*_NW_ denotes the width of the superconducting nanowire, while *w*_pr_ is the width of the Al probe. The tunnel resistance *R*_T_, the maximum absolute values of the flux-to-current (|*dI*/*d*Φ|_Max_) and flux-to-voltage (|*dV*/*d*Φ|_Max_) transfer functions are measured at *T*_bath_ = 25 mK.

Sample	*L* (nm)	*w*_NW_ (nm)	*w*_pr_ (nm)	*R*_T_ (kΩ)	|*dI*/*d*Φ|_Max_ (nA/Φ_0_)	|*dV*/*d*Φ|_Max_ (mV/Φ_0_)
A	150	60	30	56	12.0	0.52
B	140	65	30	61	10.5	0.49
C	155	50	40	65	8.3	0.48
D	150	45	35	36	5.1	0.19

### Transport spectroscopy

The SQUIPT operation relies on the magnetic-flux control of the weak-link DOS. For ideal wire/ring interfaces, the minigap *ε*_G_ in the middle of the wire in the short junction limit, i.e., when the interelectrode spacing *L* is shorter than the diffusive coherence length $L\le \sqrt{\hslash D/{{\rm{\Delta }}}_{{\rm{R}}}}$, is *ε*_G_ = Δ_R_|cos(*φ*/2)|. Here, $\hslash$ is the reduced Planck constant, *D* is the diffusion coefficient of the nanowire, and Δ_R_ is the energy gap of the ring. In the limit of negligible geometric and kinetic inductance of the ring compared to the weak-link kinetic inductance, the fluxoid quantization imposes *φ* = 2*π*Φ/Φ_0_ where Φ is the external magnetic flux piercing the loop. As a result, the electric transport through the leads is Φ_0_-periodic with the flux of the applied magnetic field. Thus the SQUIPT acts as an interferometer. All the measurements are performed in a ^3^He/^4^He dilution refrigerator. The evolution of the electrical transport through the devices with the magnetic field is periodic with a period of *B*_0_ = 3.3 G. The corresponding area *A*_eff_ for magnetic field penetration is ${A}_{{\rm{e}}{\rm{f}}{\rm{f}}}={{\rm{\Phi }}}_{0}/{B}_{0}\simeq 6\,\mu {\text{m}}^{2}$, consistent with the size of the devices.

**Figure 2:**
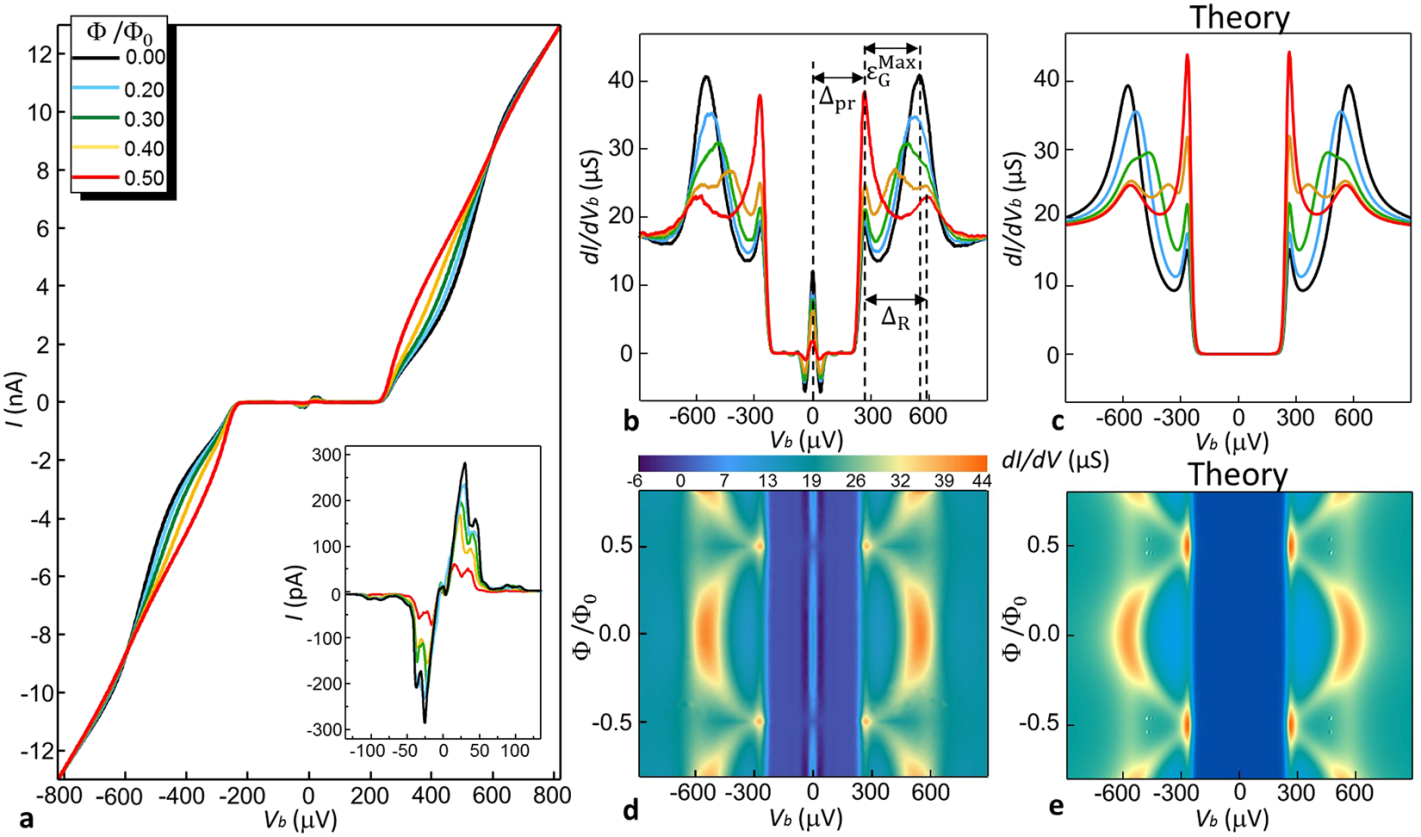
Interferometer characterization at *T*_bath_ = 25 mK (sample-A). (**a**) Current-voltage *I*(*V*_b_) characteristics measured for some values of the flux Φ generated by the external magnetic field. Inset: enlargement around zero bias of the *I*(*V*_b_) characteristics. The peak around zero bias, with maximum magnitude $I\simeq$ 280 pA, is the Josephson current flowing through the superconducting probe junction. (**b**) Measured and (**c**) theoretical differential conductance as function of voltage bias for Φ values as in (**a**). (**d**) Experimental and (**e**) theoretical color plot of the differential conductance *dI*/*dV* versus voltage and magnetic flux.

The characterization of the device (sample-A) at base temperature *T*_bath_ = 25 mK is displayed in Fig. 2. Figure 2(a) shows the *I*(*V*_b_) characteristics of the device measured for different values of the applied magnetic flux. Here we can identify four regions: (i) |*V*_b_| ≤ 250 *μ*V: the current is strongly suppressed and the phase modulation is negligible, ii) 250 *μ*V ≤ |*V*_b_| ≤ 600 *μ*V: the current increases significantly and a modulation with respect to the applied magnetic field is clearly visible, iii) |*V*_b_| ≥ 600 *μ*V: a crossing point of the current-voltage characteristics and a small modulation is still visible, iv) at higher voltage the curves approach the ohmic behaviour. Moreover, since the probe is superconducting, the tunnel junction supports a supercurrent, due to the Josephson effect. This is shown in the inset of Fig. 2(a), where a magnification at low voltage is displayed. The supercurrent is ~ 280 pA at Φ = 0, where the minigap is maximum, while its value is reduced down to ~ 60 pA at Φ/Φ_0_ = 0.5, showing a ~ 80% suppression with respect to the zero-flux value of the supercurrent. This behaviour gives an additional demonstration of the weak-link strong modulation of the minigap^23^.

The magnetic field modulation of the nanowire DOS it is better visualized by considering the evolution of the differential conductance in the flux, as displayed in Fig. 2(b). The curves are obtained through numerical differentiation of the current-voltage characteristic shown in panel (a). Following to the *I*(*V*_b_) characteristics, the conductance is strongly suppressed for |*V*_b_| ≤ 250 *μ*V, except for the structures related to the Josephson effect. At ~250 *μ*V an abrupt increase in the current [see Fig. 2a] results in a conductance peak, whose intensity is enhanced by the applied magnetic flux. At higher absolute voltage values the peak evolution is more complex. At zero flux additional conductance peaks are visible at |*V*_b_| = 550 *μ*V. By increasing the magnetic flux, these peaks move toward smaller absolute voltages and their intensities become smaller, revealing the presence of additional structures at 580 *μ*V.

**Figure 3:**
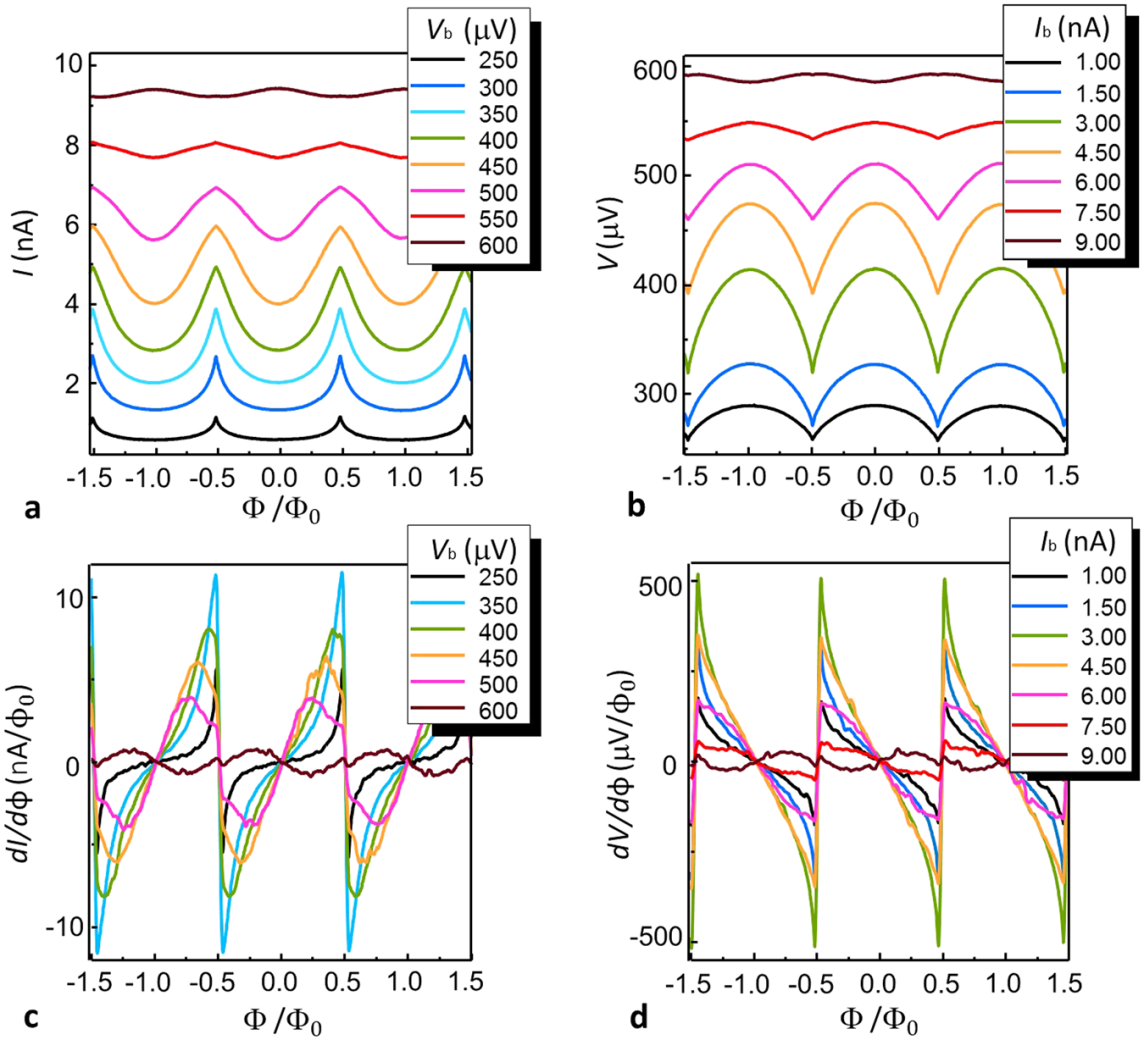
Interferometric behaviour of the V-based SQUIPT (sample-A) at *T*_bath_ = 25 mK. (**a**) Current modulation *I*(Φ) for different values of the bias voltage *V*_b_ applied to the tunnel junction. (**b**) Voltage modulation curves *V*(Φ) at different values of the bias current *I*_b_ through the junction. (**c**) and (**d**) Flux-to-current *dI*/*d*Φ and flux-to-voltage *d* *V*/*d*Φ transfer functions, obtained differentiating *I*(Φ) and *V*(Φ), respectively.

We explain this behaviour as follows. For simplicity, we consider only the quasiparticle contribution to the electrical current. The current through the probe/weak-link tunnel junction is given by ref. 35

1 $$I=\frac{1}{e{R}_{{\rm{T}}}}{\int }_{-\infty }^{+\infty }dE\frac{1}{{w}_{{\rm{pr}}}}{\int }_{{x}_{0}-\frac{{w}_{{\rm{pr}}}}{2}}^{{x}_{0}+\frac{{w}_{{\rm{pr}}}}{2}}dx{N}_{{\rm{NW}}}(E,{\rm{\Phi }},x){N}_{{\rm{pr}}}(E-eV)[\,f(E-eV)-f(E)]$$

where −*e* is the electron charge, *R*_T_ is the normal-state resistance of the junction, *E* is the quasiparticle energy with respect to the chemical potential and *V* is the voltage across the junction. Here *N*_pr_ and *N*_NW_ are the normalized DOS functions of the probe and the nanowire, respectively. Since ${w}_{{\rm{pr}}}/L\sim 0.2$, we approximate $\frac{1}{{w}_{{\rm{pr}}}}{\int }_{{x}_{0}-\frac{{w}_{{\rm{pr}}}}{2}}^{{x}_{0}+\frac{{w}_{{\rm{pr}}}}{2}}dx{N}_{{\rm{NW}}}(E,{\rm{\Phi }},x)\approx {N}_{{\rm{NW}}}\,(E,{\rm{\Phi }},{x}_{0})$, in order to simplify the calculation (see Supplementary Information). The system is assumed to be at thermal equilibrium at temperature *T*_bath_, thus the states population is expressed by the Fermi-Dirac distribution *f*(*E*) = (${e}^{F/{k}_{{\rm{b}}}{T}_{\mathrm{bath}}}$ + 1)^−1^. The superconducting probe DOS is *N*_pr_(*E*) = *N*_BCS_(*E*, Γ_pr_, Δ_pr_), where ${N}_{{\rm{BCS}}}(E,{\rm{\Gamma }},{\rm{\Delta }})=|{\rm{Re}}[\frac{E+i{\rm{\Gamma }}}{\sqrt{{(E+i{\rm{\Gamma }})}^{2}-{{\rm{\Delta }}}^{2}}}]|$ is the BCS DOS, smeared by a finite Dynes parameter Γ^36^. The nanowire DOS is affected by the proximity effect, which is properly described by the quasiclassical Usadel equations for diffusive systems^37, 38^. In the short junction limit the solution for the DOS can be obtained analytically^39^

2 $${N}_{{\rm{N}}{\rm{W}}}(E,{\rm{\Phi }},{x}_{0})=|{\rm{R}}{\rm{e}}[\frac{E+i{{\rm{\Gamma }}}_{{\rm{R}}}}{\sqrt{{(E+i{{\rm{\Gamma }}}_{{\rm{R}}})}^{2}-{{\rm{\Delta }}}_{{\rm{R}}}^{2}{\cos }^{2}(\pi {\rm{\Phi }}/{{\rm{\Phi }}}_{0})}}\cosh (\frac{2{x}_{0}}{L}{\cosh }^{-1}\sqrt{\frac{(E+i{{\rm{\Gamma }}}_{{\rm{R}}}{)}^{2}-{{\rm{\Delta }}}_{{\rm{R}}}^{2}{\cos }^{2}(\pi {\rm{\Phi }}/{{\rm{\Phi }}}_{0})}{{(E+i{{\rm{\Gamma }}}_{{\rm{R}}})}^{2}-{{\rm{\Delta }}}_{{\rm{R}}}^{2}}})]|,$$

where the ring is modeled as an effective BCS superconductor with pairing potential Δ_R_ and Dynes parameter Γ_R_. This expression simplifies in the limit of a perfectly centered probe (*x*_0_ = 0), namely *N*_NW_(*E*, Φ, 0) = *N*_BCS_(*E*, Γ_R_, *ε*_G_(Φ)), where ε_G_(Φ)=Δ_R_|cos(πΦ/Φ_0_)| is the flux-dependent minigap induced in the nanowire DOS. Similar applies at Φ = 0, where the wire DOS is independent on the probing position *N*_NW_(*E*, 0, *x*_0_) = *N*_BCS_(*E*,Γ_R_,Δ_R_). Notably, even for Φ = 0.5 Φ_0_, the DOS retains a non-trivial dependence on the energy *E* if *x*_0_ ≠ 0.

Despite its simplicity, the model captures the main features observed in the differential conductance curves, including the evolution of the various peaks. The parameters Δ_pr_, Δ_R_, Γ_pr_, Γ_R_, *x*_0_, *R*_T_ of the model can be separately estimated thanks to the rich structure expressed by the experimental curves. The normal state resistance of the tunnel junction is easily extracted from the *I*(*V*_b_) characteristic in the ohmic limit (where $I\sim {V}_{{\rm{b}}}/{R}_{{\rm{T}}}$) as ${R}_{{\rm{T}}}\sim 56\,{\rm{k}}{\rm{\Omega }}$. In the region $|{V}_{{\rm{b}}}|\le {{\rm{\Delta }}}_{{\rm{pr}}}/e\simeq 250\,\mu {\rm{V}}$ the conductance is strongly suppressed due to the superconducting energy gap in the probe DOS, whereas at higher voltages the conductance is large. This feature allows us to estimate both the Al probe pairing potential ${{\rm{\Delta }}}_{{\rm{pr}}}\simeq 255\,\mu e{\rm{V}}$ and the ring Dynes parameter ${{\rm{\Gamma }}}_{{\rm{R}}}\sim 0.35\,{{\rm{\Delta }}}_{{\rm{R}}}$. Consequently, the Al probe Dynes parameter ${{\rm{\Gamma }}}_{{\rm{pr}}}\sim {10}^{-3}\,{{\rm{\Delta }}}_{{\rm{pr}}}$ is determined from the small subgap conductance $\sim {{\rm{\Gamma }}}_{{\rm{R}}}{{\rm{\Gamma }}}_{{\rm{pr}}}/{{\rm{\Delta }}}_{{\rm{R}}}{{\rm{\Delta }}}_{{\rm{pr}}}{R}_{{\rm{T}}}$. The peaks at voltage $|{V}_{{\rm{b}}}|=({{\rm{\Delta }}}_{{\rm{pr}}}+{{\rm{\Delta }}}_{{\rm{R}}})/e\simeq 580\,\mu {\rm{V}}$ which are visible at Φ = 0.5 Φ_0_ allows for an estimation of the bilayer effective pairing potential ${{\rm{\Delta }}}_{{\rm{R}}}\simeq 310\,\mu e{\rm{V}}$. Furthermore, it reveals a decentering in the probe position, which is set to *x*_0_ = 0.25 *L*, and is consistent with the scanning electron micrograph displayed in the enlarged view of the weak-link of Fig. 1b). Summarizing, the three peaks structure at increasing voltage reside approximately at $e|{V}_{{\rm{b}}}|\simeq {{\rm{\Delta }}}_{{\rm{pr}}},\,e|{V}_{{\rm{b}}}|\simeq {\varepsilon }_{{\rm{G}}}({\rm{\Phi }})+{{\rm{\Delta }}}_{{\rm{pr}}},\,e|{V}_{{\rm{b}}}|\simeq {{\rm{\Delta }}}_{{\rm{R}}}+{{\rm{\Delta }}}_{{\rm{pr}}}$.

The theoretical curves for the differential conductance obtained using the above parameters are shown in Fig. 2(c). An extended comparison is presented in Fig. 2(d,e), where the color plots of the experimental and theoretical differential conductance are displayed, respectively. Note that the maximum minigap value ${\varepsilon }_{{\rm{G}}}^{{\rm{Max}}}$ is slightly smaller than the ring pairing potential Δ_R_, namely ${\varepsilon }_{{\rm{G}}}^{{\rm{Max}}}\simeq 300\,\mu e{\rm{V}}$. This can be related to nonidealities in the interface between the ring/weak-link contacts.

Two facts deserve discussion. First, it is difficult to give a precise estimate of the suppression of the minigap in the nanowire DOS at 0.5 Φ_0_ due to the presence of the probe pairing potential, which masks any possible small contribution around $|{V}_{{\rm{b}}}|={{\rm{\Delta }}}_{{\rm{pr}}}/e\simeq 255\,\mu e{\rm{V}}$. Anyway, the strong flux-modulation of the tunnel probe supercurrent and the good agreement with the short-limit junction model confirm an almost full closure of the minigap. We also note that the incomplete suppression of the Josephson current at Φ = 0.5Φ_0_ could stem from the decentering of probe junction. Despite this inconvenience, the choice of a superconducting probe is beneficial for improved magnetic sensor performance, as already shown in Al-based SQUIPTs^23, 24^. Second, the large broadening parameter for the ring DOS Γ_R_ must be regarded as an effective parameter in this simplified description. In particular the origin of such large conductance is likely related to the presence of vanadium. Similar high subgap conductances have been observed in several occasions in V-based tunnel junctions^40–44^, as well as in Nb-based tunnel junctions^45^.

### Magnetic sensing performance

Here we investigate the interferometric behaviour of the sample-A SQUIPT at base temperature *T*_bath_ = 25 mK. For this purpose, we consider either the current modulation *I*(Φ) at fixed bias voltage *V*_b_ and the voltage modulation *V*(Φ) at given input current *I*_b_. The results are reported in Fig. 3.

Panel 3(a) shows the current *I*(Φ) for several values of *V*_b_ in the range from 250 *μ*V to 600 *μ*V, where the modulation is stronger. In accordance with the curves displayed in Fig. 2(a), the shape of *I*(Φ) and the size of the modulation strongly depend on the bias voltage *V*_b_. The maximum current modulation *δI*_Max_ in a period is approximately equal to 2 nA around *V*_b_ = 400 *μ*V. Note the change of concavity, i.e. the current decreases for increasing magnetic field in the range [*n*Φ_0_, (*n* + 1/2)Φ_0_] (*n* is an integer number), when *V*_b_ exceeds the crossing point of the current-voltage characteristic [see Fig. 2(a)].

In this configuration, the SQUIPT acts as a flux-to-current transducer. An important figure of merit for magnetic field sensing applications is the flux-to-current transfer function, namely *dI*/*d*Φ. The curves obtained by numerical differentiation of the experimental data shown in Fig. 3(a) for six different values of bias voltage around the optimum working point are shown in 3(c). The transfer function exhibits the maximum value of |*dI*/*d*Φ|_Max_ ≅ 12 nA/Φ_0_ at *V*_b_ =350 μV. A similar analysis is repeated for the current bias configuration. Figures 3(b) and (d) show the voltage modulation *V*(Φ) and the flux-to-voltage transfer function *dV*/*d*Φ for some values of the bias current *I*_b_ in the range [1 nA, 9 nA], respectively. In the half period [0, 0.5 Φ_0_] the voltage diminishes with the magnetic field due to the shrinking of the energy gap in the nanowire DOS, except for currents bigger than 8 nA, where the opposite occurs. The maximum voltage modulation *δV*_Max_ and the maximum flux-to-voltage transfer function |*dV*/*d*Φ|_Max_ is obtained at 3.0 nA and reaches values as high as *δV*_Max_ ≅ 100 μV and |*dV*/*d*Φ|_Max_ ≅ 520 μV/Φ_0_, respectively.

Another relevant figure of merit for a magnetometer is the noise-equivalent flux (NEF) or flux sensitivity (Φ_NS_), which gives the amount of noise per output bandwidth, and it is commonly expressed in units ${{\rm{\Phi }}}_{0}/\sqrt{{\rm{H}}{\rm{z}}}$. Thanks to the intermediate value of the tunnel junction resistance, the devices can efficiently operate either with voltage amplification under DC current bias or with current amplification under DC voltage bias.

In the bias current configuration the flux sensitivity is expressed by ${{\rm{\Phi }}}_{{\rm{NS}}}=\sqrt{{{\rm{S}}}_{{\rm{V}}}}/|dV/d{\rm{\Phi }}|$ where *S*_V_ is the voltage noise spectral density. The intrinsic noise in the device is mainly given by the shot noise in the probe junction and it is expressed in the zero temperature limit by $\sqrt{{S}_{{\rm{V}}}}={R}_{{\rm{d}}}\sqrt{2eI}$ where *R*_d_  = ∂*V*/∂*I* is the differential resistance at the operating point. The extrinsic noise is the input-referred noise power spectral density of the preamplifier used in this setup (NF Corporation model LI-75A, with $\sqrt{{S}_{{\rm{V}}}}\simeq 1.5\,\text{mV}/\sqrt{{\rm{H}}{\rm{z}}}$). At the optimal bias point ${I}_{{\rm{b}}}\sim 3\,{\rm{nA}}$, ${R}_{{\rm{d}}}\simeq 50\,{\rm{k}}{\rm{\Omega }}$ and the intrinsic and extrinsic noises give approximately the same contributions, reading ${{\rm{\Phi }}}_{{\rm{NS}}}\sim 3\,\mu {{\rm{\Phi }}}_{0}/\sqrt{{\rm{Hz}}}$ and ${\Phi }_{{\rm{NS}}}\simeq 2.9\,\mu {{\rm{\Phi }}}_{0}/\sqrt{{\rm{Hz}}}$, respectively.

Improved performances are obtained in the voltage bias configuration, where the intrinsic noise is reduced to ${\Phi }_{{\rm{NS}}}=\sqrt{2eI}/|dI/d{\rm{\Phi }}{|}_{{\rm{Max}}}\simeq 2.6\mu {{\rm{\Phi }}}_{0}/\sqrt{{\rm{Hz}}}$, where $I\sim 3\,{\rm{nA}}$. In this configuration, the extrinsic contribution of the current preamplifier (DL Instruments model 1211, with current spectral density noise $\sqrt{{S}_{\text{I}}}$ = 5 fA/$\sqrt{\text{Hz}}$) can be disregarded. In both configurations, the quantum limited noise ${{\rm{\Phi }}}_{\text{NS,q}}=\sqrt{\hslash {L}_{\text{g}}}\sim 10\,{\text{n}{\rm{\Phi }}}_{0}/\sqrt{{\rm{H}}{\rm{z}}}$ is negligible for typical ring geometric inductances ${L}_{{\rm{g}}}\sim 5\,{\rm{pH}}$.

### Impact of the bath temperature

**Figure 4:**
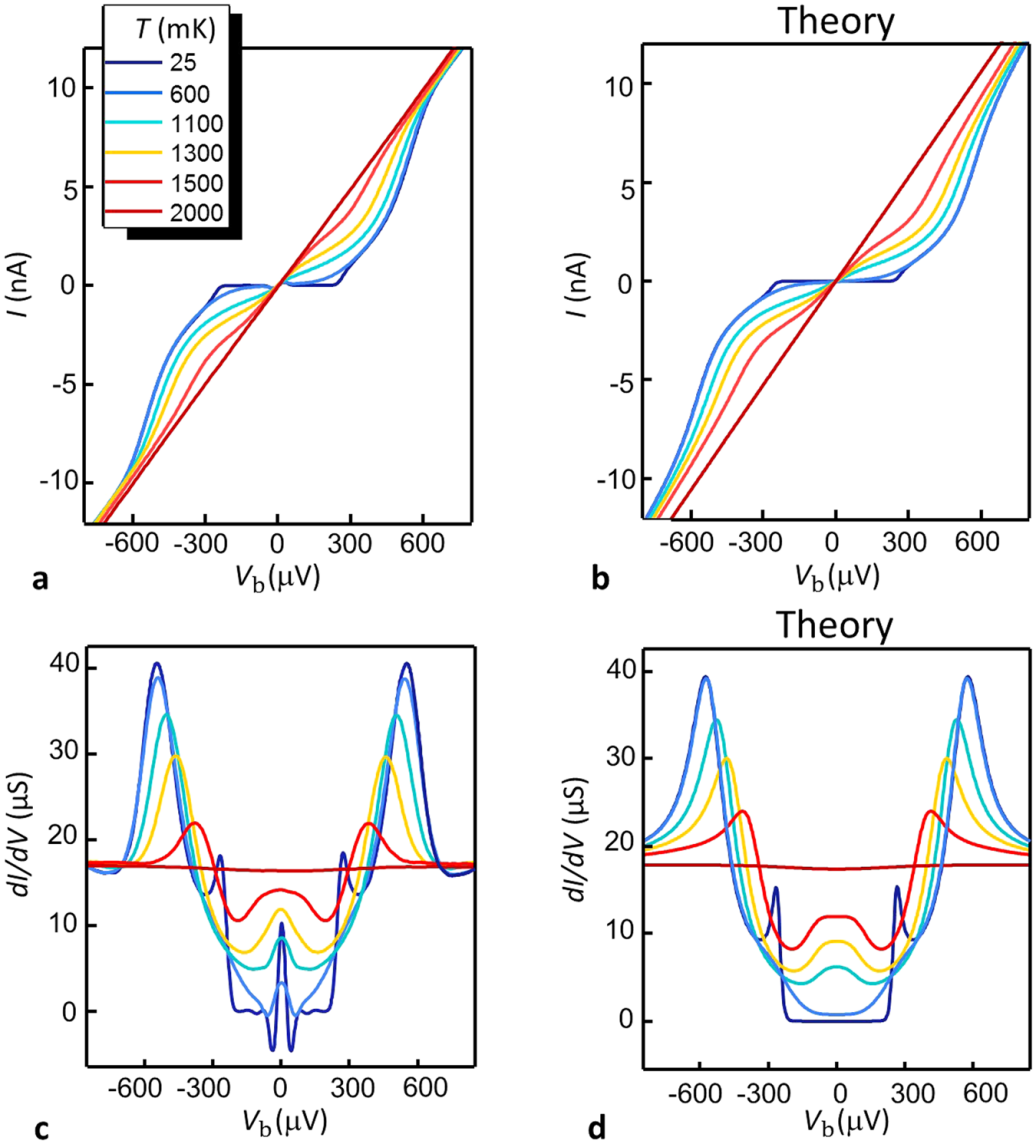
Temperature behaviour of the electrical trasport in the V-based SQUIPT. (**a**) Current-voltage *I*(*V*_b_) characteristic curves measured at Φ = 0 for several increasing temperatures of the V-SQUIPT (sample-A). (**b**) Theoretical prediction for the curves shown in (a), obtained through a numerical calculation based on the model presented in the text. (**c**) and (**d**) Measured and calculated differential conductance *dI/dV* vs voltage bias for the same temperatures as in panel (a).

The role of the temperature *T* is summarized in Fig. 4. Panel 4(a) shows the *I*(*V*_b_) current-voltage characteristics at several bath temperatures for Φ = 0 (i.e., when the induced minigap is maximum). The corresponding theoretical curves are displayed in Fig. 4(b) for the parameters extracted from the base temperature characterization, and assuming a pure BCS dependence both for Δ_pr_ and Δ_R_. As expected, the current increases with the temperature due to the broadening of the Fermi distributions and the shrinking of the probe Δ_pr_ and the ring Δ_R_ superconducting pairing potentials (hence the reduction of the minigap in the nanowire DOS *ε*_G_). Furthermore, the nonlinear behaviour of the *I*(*V*_b_) curves progressively decreases with the increase of the temperature, showing an almost linear characteristic around 2 K, when the superconducting features disappear. This value is consistent with the critical temperature extracted from the ring pairing potential Δ_R_, namely ${T}_{{\rm{c}},{\rm{r}}{\rm{i}}{\rm{n}}{\rm{g}}}={{\rm{\Delta }}}_{{\rm{R}}}/1.764{k}_{{\rm{b}}}\simeq 2\,{\rm{K}}$.

A deeper insight comes from the analysis of the differential conductances. Figure 4(c) shows the curves obtained by numerical differentiation of the experimental data, while the corresponding theoretical curves are plotted in Fig. 4(d). Compared to the previously discussed features [see Fig. 2(b) and (c)], the peak structures have an easier identification, since the probe position *x* has no effect on the nanowire density of states at Φ = 0, i.e., *N*_NW_(*E*, 0, *x*) = *N*_BCS_(*E*, Γ_R_, Δ_R_). At low temperature, i.e. for $T\ll {T}_{{\rm{c}},{\rm{p}}{\rm{r}}{\rm{o}}{\rm{b}}{\rm{e}}}={{\rm{\Delta }}}_{{\rm{p}}{\rm{r}}}/1.764{k}_{\text{b}}\simeq 1.67\,{\rm{K}}$ the Josephson supercurrent contribution appears as a low voltage peak. By increasing the temperature, this contribution becomes small due to the reduction of the probe and ring pairing potentials. In addition, it is masked by the quasiparticle contribution at low voltage, which becomes significant due to Fermi distribution broadening, thence it is hardly spotted already at 1.1 K.

The peaks at $|{V}_{{\rm{b}}}|={{\rm{\Delta }}}_{{\rm{p}}{\rm{r}}}/e\simeq 255\,\mu {\rm{V}}$ are smoothed out by the thermal broadening already at *T* = 600 mK, where only the peaks at higher absolute voltage are detectable. As discussed before, the size of the minigap is related to the position of these peaks, which reside at |*V*_b_| = (*ε*_G_ (Φ) + Δ_pr_)/*e*. As expected, their intensity decreases by increasing the temperature and they shift toward smaller absolute voltages, confirming the shrinking of the minigap, especially above $900\,{\rm{mK}}\simeq 0.4\,{{\rm{T}}}_{{\rm{c}},\mathrm{ring}}$, according to the usual BCS dependence of the superconducting pairing potential. When the temperature reaches the ${T}_{\text{c},{\rm{r}}{\rm{i}}{\rm{n}}{\rm{g}}}\simeq 2$ K, the differential conductance becomes almost flat.

**Figure 5:**
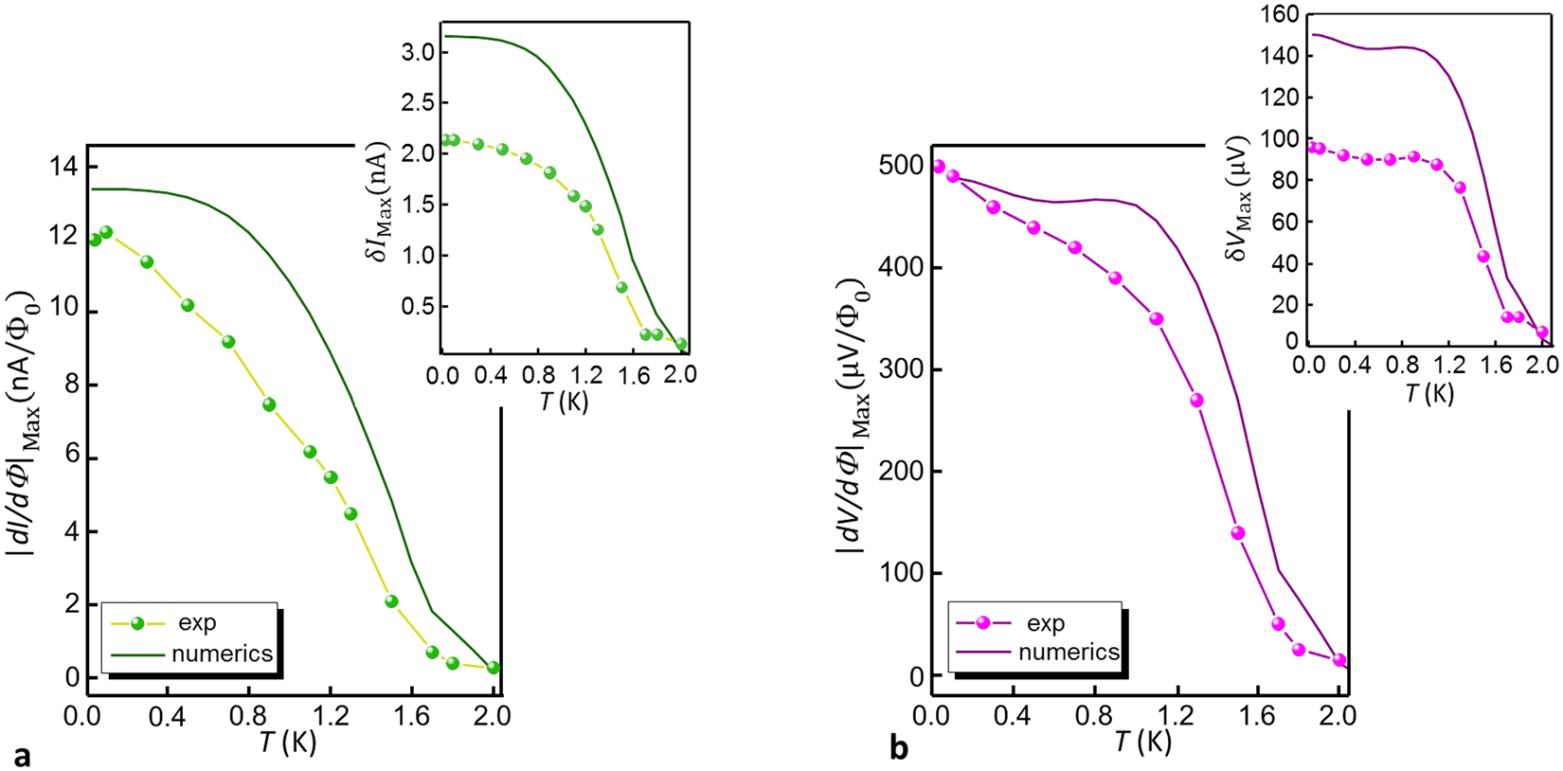
Temperature evolution of the magnetometer performance. (**a**) and (**b**) Temperature dependence of the maximum absolute value of the flux-to-current (|*dI*/*d*Φ|_Max_) and flux-to-voltage (|*dV*/*d*Φ|_Max_) transfer function, respectively. The insets show the maximum peak-to-peak current amplitude (*δI*_Max_) and the maximum peak-to-peak voltage amplitude (*δV*_Max_), respectively. The lines connecting experimental data are guides to the eye. The theoretical predictions based on the simplified model are also plotted (solid lines).

The temperature evolution of the interferometric performance of the device is shown in Fig. 5. We already discussed how temperature increases the quasiparticle current, due to the thermal broadening and the shrinking of the probe and the ring gap. This smearing unavoidably influences the magnetic flux dependence of the current at fixed voltage and reduces the SQUIPT performance. This aspect is shown in Fig. 5(a), where the maximum amplitude of the flux-to-current transfer function is displayed (points with line). This quantity decreases quite linearly with the temperature. Notably, the V-SQUIPT still exhibits a large sensitivity $|dI/d{\rm{\Phi }}{|}_{{\rm{Max}}}\simeq 2\,{\mathrm{nA}/{\rm{\Phi }}}_{0}$ at 1.5 K. This represents a relevant improvement with respect to the previous SQUIPT devices, where similar values were only possible below 1 K. In the inset, the maximum current modulation at fixed voltage is plotted against temperature, showing a swing $\delta {I}_{{\rm{Max}}}\simeq 700$ pA at 1.5 K. Similar considerations apply for the maximum flux-to-voltage |*dV*/*d*Φ|_Max_ and maximum voltage modulation *δV*_Max_ at fixed current bias. The results are reported in Fig. 5(b). These curves decrease slowly for temperatures $T\le 0.4{T}_{\text{c},{\rm{r}}{\rm{i}}{\rm{n}}{\rm{g}}}\simeq 0.9$ K and then drop when the temperature approaches the bilayer critical temperature. As discussed before, the performances at 1.5 K are still remarkable, with a maximum transfer function $|dV/d{\rm{\Phi }}{|}_{{\rm{M}}{\rm{a}}{\rm{x}}}\simeq 140\,\mu {\rm{V}}{/{\rm{\Phi }}}_{0}$ and a maximum swing $\delta {V}_{{\rm{Max}}}\simeq 40\,\mu {\rm{V}}$. In the plot the theoretical prediction, according to the simplified model used throughout the paper, are also reported (solid lines). The temperature evolution of the maximum flux-to-current (flux-to-voltage) transfer functions are similar to the experimental result, whereas significant deviations are observed in the current (voltage) swing. Our simplified model gives a somewhat less satisfactory fit for the current *I*(Φ) and voltage *V*(Φ) magnetic flux dependence than for the differential conductance (see Supplementary Information).

## Discussion

**Figure 6:**
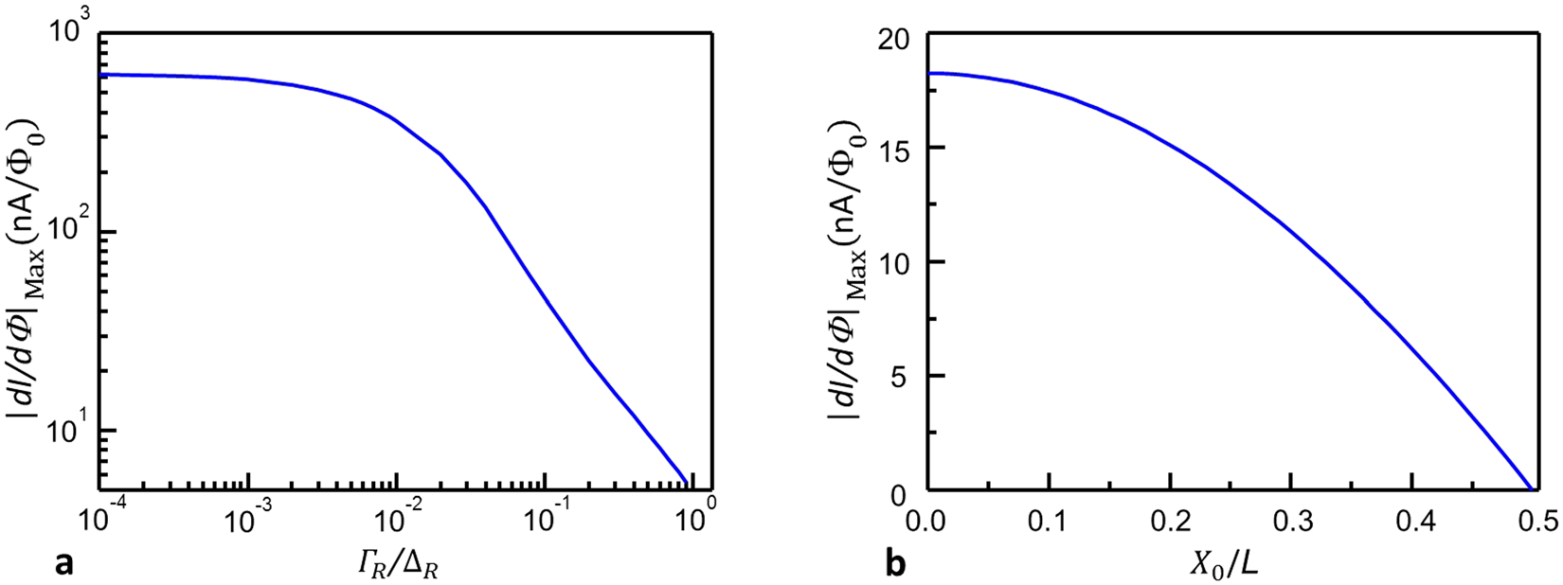
Theoretical prediction of the parameters impact on interferometers performance. (**a**) Maximum absolute value of the flux-to-current transfer function (|*dI*/*d*Φ|_Max_) versus normalized Dynes parameters of the ring. (**b**) Maximum absolute value of the flux-to-current transfer function (|*dI*/*d*Φ|_Max_) versus the probe position respect to the middle of the nanowire.

In summary, we have presented the fabrication and characterization of V-based SQUIPTs realized with a V-Al bilayer ring. Our quantum interference proximity transistors show good magnetometric performance with low noise-flux sensitivity down to $\sim 2.6\,\mu {{\rm{\Phi }}}_{0}/\sqrt{{\rm{Hz}}}$ at base temperature *T*_bath_ = 25 mK. Previously, higher interferometric performances have been reported for Al-based SQUIPTs (magnetic flux resolution as low as $500\,{{\rm{n}}{\rm{\Phi }}}_{0}/\sqrt{{\rm{Hz}}}$ ^23^. From the theoretical side, the V-based SQUIPT should guarantee better performance compared to the Al-based technology, since the predicted flux-to-voltage transfer function scales as Δ_R_/*e* ^21^. This is not observed due to the high subgap conductance of the superconducting vanadium, which limits the device performance. This is displayed in the first panel of Fig. 6, where the theoretical maximum flux-to-current transfer function is plotted as a function of the ring Dynes parameter. A smaller role is played also by the decentering of the probe respect to the middle of the nanowire, as shown in Fig. 6(b). The origin of the large subgap conductance of vanadium is still not understood and may be related to the evaporation process. Despite this, the use of vanadium allows us to obtain unprecedented performances in terms of maximum operating temperature ($T\simeq 2.0\,{\rm{K}}$), since the Al-based SQUIPTs typically work only up to ~1 K. Furthermore, our interferometers still exhibit high sensitivity ($|dI/d{\rm{\Phi }}{|}_{{\rm{Max}}}\simeq 2\,{\rm{nA}}/{{\rm{\Phi }}}_{0}$ and $|dV/d{\rm{\Phi }}{|}_{{\rm{M}}{\rm{a}}{\rm{x}}}\simeq 140\,\mu {\text{V}/{\rm{\Phi }}}_{0}$) at 1.5 K. The main features in our devices are well reproduced within a simplified theoretical model. The V-based SQUIPT configuration is a proof-of-concept showing the V-Al material combination is an suitable candidate for the realization of high-performance magnetometers operating above 1 K.

Finally, furthermore improvements will be possible with the adoption of superconducting materials with wider energy-gap, such as lead, niobium and niobium nitride. This will extend the magnetometer working operation at higher temperatures, allowing SQUIPT applications at temperatures accessible with the technology of ^4^He cryostats, in order to try to compete with the state of the art nanoSQUID^46, 47^ (flux resolution $45\,{{\rm{n}}{\rm{\Phi }}}_{0}/\sqrt{{\rm{Hz}}}$).

## Methods

### Device fabrication details

The devices were fabricated through single electron-beam lithography process followed by a three-angle shadow-mask evaporation of metals through a suspended resist mask. At first, an oxidized silicon wafer was covered with a suspended bilayer resist mask (1200-nm copolymer, 250-nm polymethyl methecrylate (PMMA)) by spin-coated process. Then the device structures have been patterned onto the substrate via electron-beam lithography. The EBL step is followed by development in 1:3 mixture of MIBK:IPA (methyl isobutyl ketone:isopropanol) for typically 1 min and 30 sec, followed by a rinse in pure IPA and drying with nitrogen. Then, the sample was processed in a ultra-high vacuum (UHV) evaporator (base pressure of 10^−10^ Torr) for the metalization process. 15 nm of Al was deposited at 1.5 Å/s at an angle of *θ* = 40° to form the superconducting electrode of the probe tunnel junction. Subsequently, the sample was exposed to 100 mTorr O_2_ for 5 min to realize the tunnel barrier. Next, the sample was tilted to an angle of *θ* = 20° for the evaporation of 20 nm of Al to form the superconducting nanowire. Subsequently, 50 nm of Al was deposited at *θ* = 0° to realize the first layer of the bilayer ring. Finally, at the same angle 50 nm of V was evaporated at 3 Å/s in order to realize the upper layer of V/Al superconducting ring. The magneto-electric measurements were performed in a ^3^He/^4^He dilution refrigerator in a range temperature from 25 mK to 2 K using room-temperature preamplifiers.

## Electronic supplementary material

Supplementary Information
