# Supplementary material for: High operating temperature in V-based superconducting quantum interference proximity transistors
Source: Sci Rep. 2017 Aug 18;7:8810. doi: 10.1038/s41598-017-09036-0 (PMC5562923; doi:10.1038/s41598-017-09036-0)
Supplement: Supplementary file 1 — Supplementary Information [file 41598_2017_9036_MOESM1_ESM.pdf]

Supplementary Information: High operating temperature in V-based superconducting quantum interference proximity transistors.

Nadia Ligato, Giampiero Marchegiani, Pauli Virtanen, Elia Strambini, Francesco Giazotto

S1 Kinetic Inductance for the ring and the wire of the SQUIPT

Within the Mattis-Bardeen theory[1], the kinetic inductance $L_{\rm kin}$ of a superconducting strip with length l, width w and thickness t is given by $L_{\rm kin} = \hbar R/\pi\Delta$, where Δ is the superconducting order parameter and $R = \rho l/wt$ is the normal state resistance of the superconducting strip (here ρ is the normal state resistivity). This expression can be used to estimate the kinetic inductance of the superconducting loop of the SQUIPT when the ring consist of a single superconductor.

For a comparison, we consider a sinusoidal current-phase relation for the superconducting weak-link, which in the short junction limit is valid when the temperature is not too small compared to the critical temperature[2]. Under this assumption, the minimal kinetic inductance at zero phase bias $\phi = 0$ reads $L^{\rm NW}_{\rm kin} \approx \hbar R_{NW}/\pi\Delta$, where $R = \rho^{\rm NW} l^{\rm NW}/w^{\rm NW} t^{\rm NW}$ is the normal state resistance of the weak link.

The ratio between the kinetic inductance of the wire and the ring is

$$\frac{L^{\rm NW}_{\rm kin}}{L^{\rm R}_{\rm kin}} = \frac{\rho^{\rm NW} l^{\rm NW} t^{\rm R} w^{\rm R}}{\rho^{\rm R} l^{\rm R} t^{\rm NW} w^{\rm NW}} \tag{S1}$$

where the superscripts NW, R refer to the nanowire and ring, respectively.

The dimensions of the Al nanowire (device A) are $l^{\rm NW} = 150$ nm, $t^{\rm NW} = 20$ nm and $w^{\rm NW} = 60$ nm. We assume $\rho^{NW} \simeq 5\ \mu\Omega$ cm, which is the typical resistivity for 25 nm Al layer at 4.2 K evaporated in past experiments, consistently with the values reported in the literature[3–6]. If we consider a ring made of Al with dimensions $l^{\rm NW} = 6\ \mu$m, $t^{\rm NW} = 50$ nm and $w^{\rm R} = 1\ \mu$m and same resistivity (although in general the resistivity drops by increasing the thickness of the layer) we obtain $L^{\rm NW}_{\rm kin}/L^{\rm R}_{\rm kin} \sim 1.05$. The resistivity of the vanadium may vary quite strongly depending on evaporation conditions. Considering

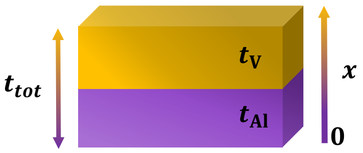

Figure S1. V-Al bilayer scheme. The strip has total thickness $t_{\rm tot} = t_{\rm V} + t_{\rm Al}$, where $t_{\rm V}$ and $t_{\rm Al}$ are the thicknesses of vanadium and aluminium, respectively.

literature values[7–11], we estimate the V layer resistivity $\rho_{\rm V}$ to range approximately from the same resistivity of the Al 5 $\mu\Omega$ cm to a value 5 times larger 25 $\mu\Omega$ cm. As a consequence, this would produce a potentially large deviation from the ideal condition $L^{\rm NW}_{\rm kin}/L^{\rm R}_{\rm kin} \gg 1$. When the superconducting ring is made of a bilayer, the situation is more involved (as we detail in the next subsection): In first approximation it is possible to model the total kinetic inductance of the bilayer as the parallel of the kinetic inductance of the two layers. This simple calculation shows how the inclusion of the Al underlayer provides a suitable geometry for the good phase biasing of the device, independently of the specific properties of the vanadium layer.

S2 Bilayer modeling

The spectral properties of the V-Al bilayer in the dirty limit can be modeled within the Usadel formalism[12]. The problem formulation is similar to the one given by Fominov and Feigel'man for the properties of a thin NS bilayer[13]. In the numerical computation we model the bilayer as a superconducting strip with total thickness $t = 100$ nm and we assume a ratio 1:1 ($t_{\rm Al} = t_{\rm V} = 50$ nm) between the two layers, accordingly to the experimental realization (Fig. 1). We assume a clean interface between the two layers.

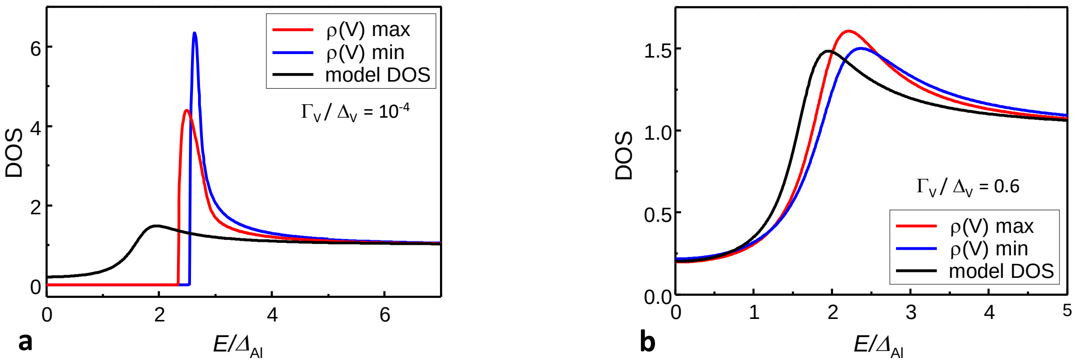

Figure S2. Density of states at the bottom of the Al layer for a 100 nm V-Al bilayer with thickness ratio 1:1. The curves for 2 different values of the vanadium resistivity are compared to the effective BCS DOS used in the text for the theoretical interpretation. Both small a) and large b) Dynes parameter in the V layer are considered.

A parameter relevant for the properties for the bilayer is

$$p = \frac{t_V \rho_{Al} D_{Al} \lambda_V}{t_{Al} \rho_V D_V \lambda_{Al}} \tag{S2}$$

where ρ_X and D_X are the normal state resistances and the diffusion constants of the materials X =Al,V (Einstein relation $D_X^{-1} = \rho_X e^2 \nu_X$ is assumed and ν_X is the density of states at the Fermi level). The coupling constants in the two superconducting layers $\lambda_X = -\ln(\Delta_X / 2E_c^X)$ depend in the weak coupling limit on the cutoff energy $E_c^X \sim k_b \theta_D^X$ where θ_D^X is the Debye temperature.

The density of states at the Fermi level $\nu_X = N_X(E_F) d_X / M_X$ are taken from the literature. Here N_X is the density of states at the Fermi level for atom ($N_{Al}(E_F) = 0.208$ eV^{-1} , $N_V(E_F) = 1.31$ eV^{-1})[14], d_X is the mass density ($d_{Al} = 2.7$ g/cm^3 , $d_V = 6.0$ g/cm^3)[15] and M_X is the atomic mass ($M_{Al} = 26.98$ u , $M_V = 50.94$ u)[16]. The Debye temperature is assumed to be the same for both materials $\theta_D^{Al} = \theta_D^V = 400$ K.

For the Al layer we choose a critical temperature equal to the bulk value $T_C^{Al} = 1.2$ K, corresponding to a zero temperature order parameter $\Delta_{Al} = 178$ μeV, and typical resistivity $\rho_{Al} = 5$ $\mu\Omega$ cm and Dynes parameter $\Gamma_{Al}/\Delta_{Al} = 10^{-4}$ obtained through electron beam evaporation.

As already stated before, the properties of the vanadium deposited through electron beam evaporation are extremely sensitive to the evaporation conditions. In accordance with the discussion in the previous section, we consider $\rho_V = 5$ $\mu\Omega$ cm and $\rho_V = 25$ $\mu\Omega$ cm as minimal and maximal resistivity in the numerical computation. Similarly apply to the critical temperature of the vanadium, which can be significantly smaller than the bulk value[17], depending on the evaporation rate. In our numerical computation we set $T_c^V = 3.5$ K, which is reasonable due to the low evaporation rate and previous realizations[18]. Finally we consider two cases for the Dynes parameter of vanadium: a very ideal situation $\Gamma_V/\Delta_V = 10^{-4}$ and an extremely leaking layer $\Gamma_V/\Delta_V = 0.6$. The latter seems to describe better the results of our experiment as we show in Fig.2, where the DOS at the bottom of the Al layer is compared to the effective BCS DOS used in the main text. In particular the resistivity of the Vanadium plays a role in the determination of the energy gap of the bilayer, but does not affect significantly the subgap density of states. In particular, the large subgap conductance observe in the experiment must be associated to an high effective Dynes parameter in the V layer even in this model. Notably, the results compare quite well with the effective BCS model used in the main text.

In this model, the kinetic inductance of the bilayer is evaluated as $L_{kin}^R = \hbar / 2eI_S'(\phi)$, where the supercurrent dispersion $I_S(\phi)$ is computed starting from the solution of the Usadel equation. An approximate expression for ultrathin layers[13] can be obtained in the Cooper limit[19], where the superconducting energy gap is homogeneous along the bilayerṪhe kinetic inductance of the ring is therefore given by the parallel of the kinetic inductances of the two layers:

$$\frac{l}{wL_{kin}} = \left(\frac{d_{Al}}{\rho_{Al}} + \frac{d_V}{\rho_V} \right) \frac{\pi \Delta_V}{\hbar} \left(\frac{\Delta_V}{\Delta_{Al}} \right)^{1/(1+p)} \tag{S3}$$

In Tab. S1, we see how the approximate expressions for the kinetic inductance compare to the values obtained through the rigorous calculation. Generally, the approximation underestimates the kinetic inductance somewhat, and does not take nonzero Dynes parameters into account.

Table S1. Kinetic inductance of the V-Al bilayer. The values computed numerically $L_{\mathrm{kin}}^{\mathrm{num}}$ are compared with the approximate expression $L_{\mathrm{kin}}^{\mathrm{approx}}$ for the numerical computation parameters chosen above.

Γ_V/Δ_V	$\rho_V\,(\mu\Omega\,\mathrm{cm})$	$L_{\mathrm{kin}}^{\mathrm{num}}$ (pH)	$L_{\mathrm{kin}}^{\mathrm{approx}}$ (pH)
10^{-4}	5	1.36	1.31
10^{-4}	25	2.54	2.19
0.6	5	2.06	1.31
0.6	25	3.67	2.19

S3 Impact of the finite width of the probe

In the main text is stated that, in order to simplify the calculation, we disregard the finite width of the probe. First, we show that the high subgap conductance observed in the differential conductance curves is not originated by the finite extension of the probe. In Fig. S3, panel a) we compare the effective DOS used in the main text with the DOS obtained after averaging over the probe width $<N>=\frac{1}{w}\int_{x_0-w/2}^{x_0+w/2}N(E,\Phi,x)dx$, where we set an ideal Dynes parameter $\Gamma_R/\Delta_R=10^{-3}$. The subgap conductance in the latter case is too small to explain the experimental results. Then we quantify the relative deviation between the simplified expression $N(E,\Phi,x_0)$ and the integrated expression $<N>$ through the figure of merit

$$\delta N(E)=\frac{1}{N(E,\Phi,x_0)}\left|N(E,\Phi,x_0)-<N>\right|. \tag{S4}$$

In Fig.S3 panel b) we plot this function for different values of $\Phi\neq 0$ (there is no deviation at $\Phi=0$). We see that the maximum relative deviation is always smaller or equal than 1%.

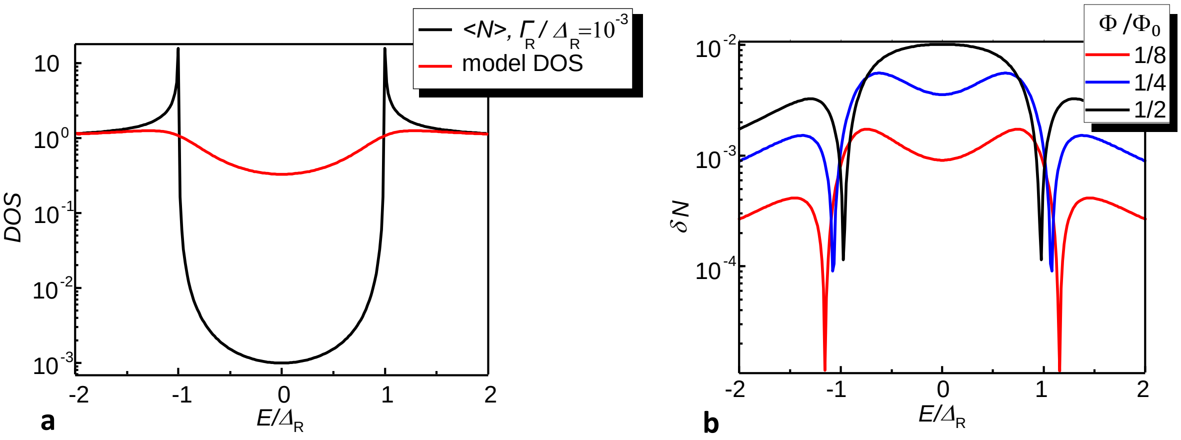

Figure S3. Impact of the finite width of the probe on the theoretical description. (a)The averaged density of states over the finite width of the probe for an ideal ring with small Dynes parameter is compared with the effective model used in the main text at $\Phi=0$. (b)Absolute value of the relative deviation of the zero-width probe approximation for the density of states induced in the nanowire VS quasiparticle energy. Parameters are $\Gamma_R/\Delta_R=0.35$, $x_0/L=0.25$ and $w=0.2L$

S4 Theoretical flux dependence

For completeness, in this section we discuss the theoretical flux dependence obtained from the theoretical model adopted throughout the main text. The plots corresponding to the panel of the Fig. 3 of the main text are displayed in Fig. S4 We note

that the comparison with the experimental data is certainly less satisfactory compared to the differential conductance curves. In particular the predicted oscillation is larger than the observed (especially at larger voltages/currents) and the curves are quite smoother around 0.5 $\Phi_0 + n\Phi_0$. Notably, despite these deviations, the maximum current-to-flux and voltage-to-flux transfer functions are close to the one observed in the experiment. This plot explain why a large deviation in the temperature evolution of the swing is observed in the theoretical curves in Fig. 5 of the main text, whereas the temperature evolution of the maximum transfer function works better.

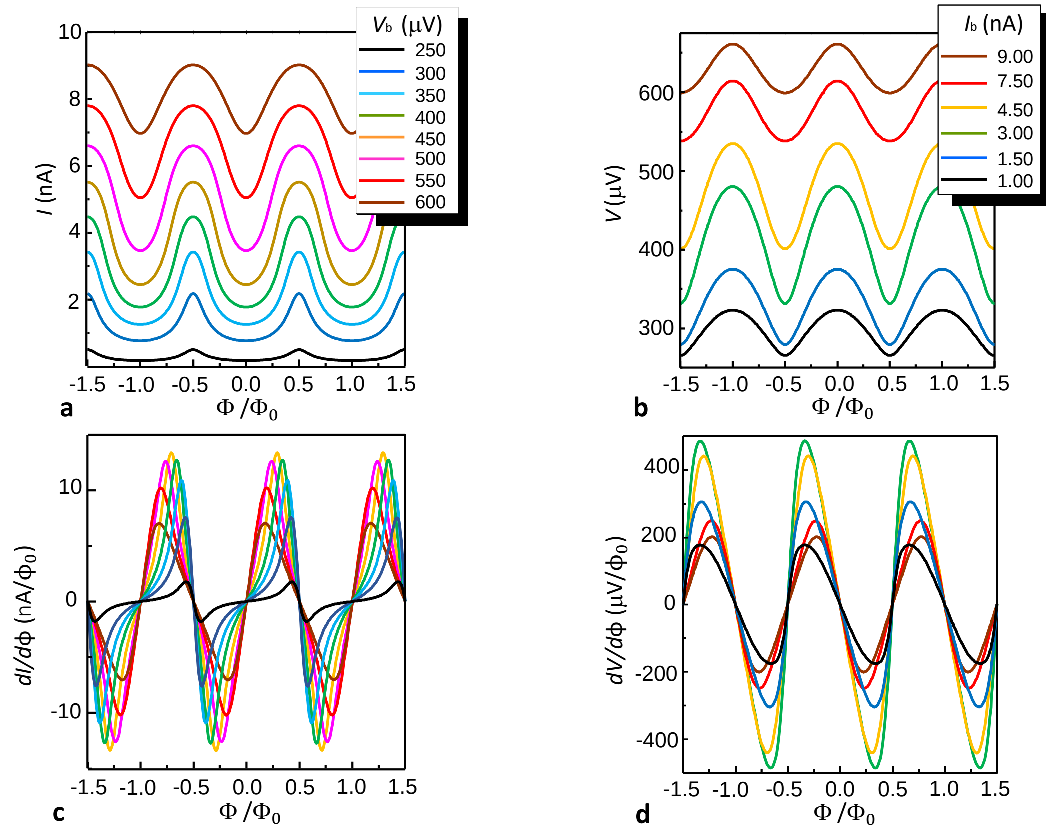

Figure S4. Theoretical interferometric behaviour. (a) Current modulation $I(\Phi)$ for different values of bias voltage V_b applied to the tunnel junction. (b) Voltage modulation curves $V(\Phi)$ at different values of the biasing current I_b through the junction. (c) and (d) Flux-to-current $dI/d\Phi$ and flux-to-voltage $dV/d\Phi$ transfer functions, obtained by numerical differentiation of $I(\Phi)$ and $V(\Phi)$, respectively.

References

1. Tinkham, M. *Introduction to Superconductivity: Second Edition*. Dover Books on Physics (Dover Publications, 2004).
2. Likharev, K. K. Superconducting weak links. *Rev. Mod. Phys.* **51**, 101–159 (1979).
3. Ullom, J. N., Fisher, P. A. & Nahum, M. Measurements of quasiparticle thermalization in a normal metal. *Phys. Rev. B* **61**, 14839–14843 (2000).
4. Courtois, H., Meschke, M., Peltonen, J. T. & Pekola, J. P. Origin of hysteresis in a proximity josephson junction. *Phys. Rev. Lett.* **101**, 067002 (2008).
5. Hübler, F., Lemyre, J. C., Beckmann, D. & v. Löhneysen, H. Charge imbalance in superconductors in the low-temperature limit. *Phys. Rev. B* **81**, 184524 (2010).
6. Peltonen, J. T., Muhonen, J. T., Meschke, M., Kopnin, N. B. & Pekola, J. P. Magnetic-field-induced stabilization of nonequilibrium superconductivity in a normal-metal/insulator/superconductor junction. *Phys. Rev. B* **84**, 220502 (2011).
7. Teplov, A. A., Mikheeva, M. N., Golyanov, V. M. & Gusev, A. N. Superconducting transition temperature, critical magnetic fields, and the structure of vanadium films. *Sov. Phys. Jetp.* **44**, 587 (1976).

8. Nicolet, M.-A. Diffusion barriers in thin films. *Thin Solid Films* **52**, 415–443 (1978).

9. Kanoda, K., Mazaki, H., Hosoito, N. & Shinjo, T. Upper critical field of v-ag multilayered superconductors. *Phys. Rev. B* **35**, 6736–6748 (1987).

10. Gibson, G. A. & Meservey, R. Evidence for spin fluctuations in vanadium from a tunneling study of fermi-liquid effects. *Phys. Rev. B* **40**, 8705–8713 (1989).

11. Aarts, J., Geers, J. M. E., Brück, E., Golubov, A. A. & Coehoorn, R. Interface transparency of superconductor/ferromagnetic multilayers. *Phys. Rev. B* **56**, 2779–2787 (1997).

12. Usadel, K. D. Generalized diffusion equation for superconducting alloys. *Phys. Rev. Lett.* **25**, 507–509 (1970).

13. Fominov, Y. V. & Feigel'man, M. V. Superconductive properties of thin dirty superconductor-normal-metal bilayers. *Phys. Rev. B* **63**, 094518 (2001).

14. McMillan, W. L. Transition temperature of strong-coupled superconductors. *Phys. Rev.* **167**, 331–344 (1968).

15. Haynes, W. *CRC Handbook of Chemistry and Physics, 97th Edition* (CRC Press, 2016).

16. Meija, J. *et al.* Atomic weights of the elements 2013 (IUPAC technical report). *Pure Appl. Chem.* **88** (2016).

17. Noer, R. J. Superconductive tunneling in vanadium with gaseous impurities. *Phys. Rev. B* **12**, 4882–4885 (1975).

18. Quaranta, O., Spathis, P., Beltram, F. & Giazotto, F. Cooling electrons from 1 to 0.4 k with v-based nanorefrigerators. *Appl. Phys. Lett.* **98**, 032501 (2011).

19. Cooper, L. N. Superconductivity in the neighborhood of metallic contacts. *Phys. Rev. Lett.* **6**, 689–690 (1961).
